# Marine Geophysical Investigation of the Chain Fracture Zone in the Equatorial Atlantic From the PI‐LAB Experiment

**DOI:** 10.1029/2018JB015982

**Published:** 2018-12-14

**Authors:** Nicholas Harmon, Catherine Rychert, Matthew Agius, Saikiran Tharimena, Tim Le Bas, J. Michael Kendall, Steven Constable

**Affiliations:** ^1^ Ocean and Earth Science University of Southampton, Waterfront Campus Southampton UK; ^2^ Now at the Jet Propulsion Laboratory California Institute of Technology Pasadena CA USA; ^3^ National Oceanography Centre Southampton UK; ^4^ School of Earth Sciences University of Bristol Bristol UK; ^5^ Scripps Institution of Oceanography University of California, San Diego San Diego CA USA

**Keywords:** Chain Fracture Zone, transform fault, Mid‐Atlantic Ridge, potential fields, gravity

## Abstract

The Chain Fracture Zone is a 300‐km‐long transform fault that offsets the Mid‐Atlantic Ridge. We analyzed new multibeam bathymetry, backscatter, gravity, and magnetic data with 100% multibeam bathymetric data over the active transform valley and adjacent spreading segments as part of the Passive Imaging of the Lithosphere Asthenosphere Boundary (PI‐LAB) Experiment. Analyses of these data sets allow us to determine the history and mode of crustal formation and the tectonic evolution of the transform system and adjacent ridges over the past 20 Myr. We model the total field magnetic anomaly to determine the age of the crust along the northern ridge segment to better establish the timing of the variations in the seafloor fabric and the tectonic‐magmatic history of the region. Within the active transform fault zone, we observe four distinct positive flower structures with several en échelon fault scarps visible in the backscatter data. We find up to −10 mGal residual Mantle Bouguer Anomaly in the region of the largest positive flower structure within the transform zone suggesting crustal thickening relative to the crustal thinning typically observed in fracture zones in the Atlantic. The extensional/compressional features observed in the Chain Transform are less pronounced than those observed further north in the Vema, St. Paul, and Romanche and may be due to local ridge segment adjustments.

## Key Points

- Variations in the seafloor fabric and crustal thickness with age suggest time variable magmatic productivity at the ridge segments
- The crustal thickness in the Chain Transform Fault does not appear thin, likely due to transpression or a high degree of serpentinization
- The transpressive features in the Chain Transform are muted compared to more northerly transforms, likely due to ridge segment adjustments

## 1 Introduction

**Figure 1:**
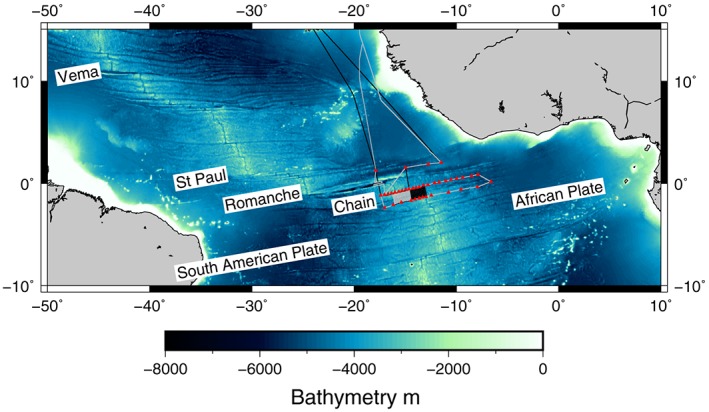
Bathymetric map of the Atlantic Ocean and the location of the PI‐LAB experiment. Long offset transforms and plates are as labeled. Red triangles show the PI‐LAB station locations near the Romanche and Chain Fracture Zones. Black line shows ship track for MGL16‐02 and gray line shows the ship track for DY072. PI‐LAB = Passive Imaging of the Lithosphere Asthenosphere Boundary.

Oceanic transform faults accommodate differential plate motions along offsets in mid‐ocean ridge segments (Sykes, 1967; Wilson, 1965) and represent an important component of plate tectonics. In slow spreading systems such as the Mid‐Atlantic Ridge (MAR), the active transform faults are thought to be zones of weakened lithosphere, which tend to form deep fracture zone valleys with reduced crustal thickness relative to the adjacent spreading segments (Blackman & Forsyth, 1991; Gregg et al., 2007; Kuo & Forsyth, 1988; White et al., 1984). Many of the fracture zones can be traced across the Atlantic Ocean basin, and the transform faults that created them appear to be long‐lived features. For instance, the Vema, St. Paul, Romanche, and Chain, which have large offsets (> 200 km), appear to have existed since the opening of the South Atlantic ~81 Ma (Figure 1; Cande et al., 1988). The sense of their ridge offsets traces the coastlines of the Gulf and Guinea of Africa and South America, and these fracture zones have been hypothesized to be initial zones of weakness during continental break up (Wilson, 1965). Understanding the structure and evolution of the active transforms of fracture zones provides insight into the long‐term behavior of the fracture zones.

Due to their long offsets, the equatorial Atlantic transform faults and fracture zones tend to amplify internal deformations associated with small plate reorganizations of the plates of the Southern Mid‐Atlantic Ocean. For example, a small degree of transtension and subsequent flexure has been suggested as the mechanism for the transverse ridge visible on 10‐ to 25‐Myr seafloor in the Vema Fracture Zone (Bonatti et al., 2005). The St. Paul Islands, which are positive flower structures that have risen above sea level, are also hypothesized to be due to a series of transtension and subsequent transpression in readjustment to the same reorganization event that uplifted Vema (Maia et al., 2016). Further to the south, the Romanche Fracture Zone exhibits a complicated morphology, with a transverse ridge similar to the Vema in the western portion of the transform fault zone (Bonatti et al., 1994). In addition, there appears to be a microplate and a southerly migration and reorientation of the transform fault over a broad zone (75 km wide) and a 10° reorientation of the eastern transform fault in the past 10 Myr (Ligi et al., 2002). Immediately to the south of the Romanche lies the Chain Fracture zone, which up until this point was largely unmapped, and thus, the visibility of these dramatic tectonic events at Chain was unknown. To date, the only bathymetric mapping of the Chain Transform was performed as part of a physical oceanography study and was not focused on understanding the tectonic history of the region (Mercier & Morin, 1997; Mercier & Speer, 1998).

Here we present the results of a comprehensive marine geophysical survey of the Chain Transform Zone to reconstruct its tectonic history over the past 20 Myr. The survey was undertaken as part of the Passive Imaging of the Lithosphere Asthenosphere Boundary Experiment (Figure 1 which was designed to image the tectonic plate [Rychert et al., 2005; Rychert & Shearer, 2009; Rychert et al., 2018a, 2018b]). In addition to deploying 39 Ocean Bottom Seismometers and Ocean Bottom Magnetotelluric instruments, we collected multibeam bathymetry and back scatter imagery, gravity and magnetics over the entire transform fault region and adjoining ridge segments. With these data we place the Chain Fracture Zone in the context of the reorganization events visible in the large offset transform faults to the north.

## 2 Data and Methods

### 2.1 Multibeam Bathymetry and Backscatter Survey

We present data from two research cruises, one aboard the RV Marcus G Langseth (MGL16‐02) in March 2016 and the other aboard the RRS Discovery (DY072) in March 2017, which were the deployment and recovery cruises for the Passive Imaging of the Lithosphere Asthenosphere Boundary experiment (Figure 1). We used the deepwater Kongsberg EM122 multibeam bathymetric surveying system aboard each ship. Within our study region we bathymetrically surveyed an area of approximately 51,200 km^2^ centered on and completely encompassing the active transform at a 50‐m resolution.

**Figure 2:**
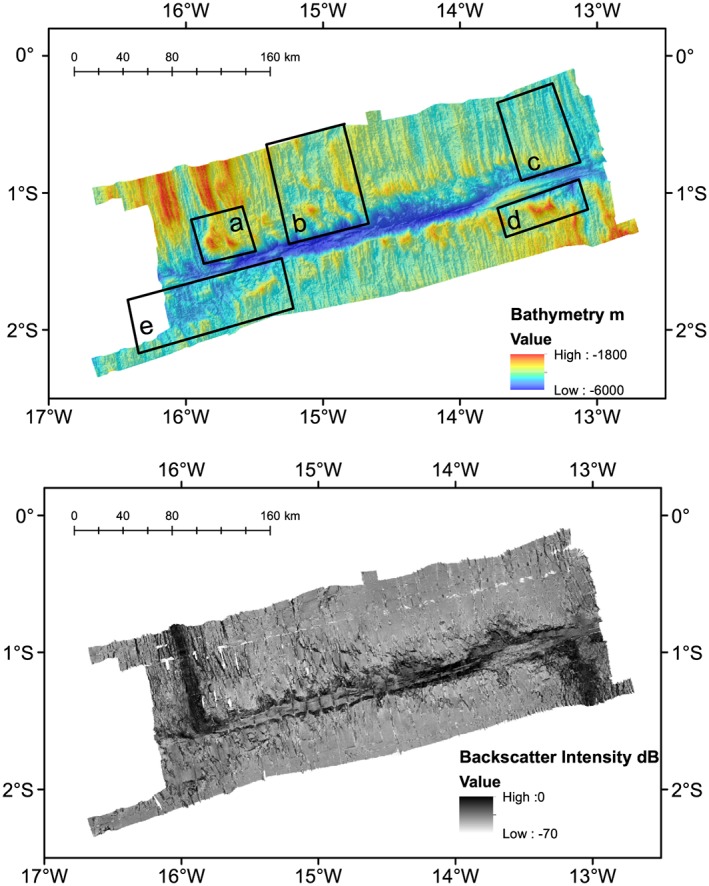
Chain Fracture Zone bathymetry and backscatter imagery at 50‐m bin size. Bathymetry is shown as background color (top) and backscatter is shown as grayscale background (bottom). Boxes a–e highlight regions shown in zoomed view in Figure 3. Zoomed views of the transform are shown in Figures 4 and 5.

Multibeam bathymetry data were processed using CARIS HIPS and SIPS software. Data were edited and filtered to remove spurious data points. Typically, only the edges of the swaths were removed, and data were very high quality. The data were then gridded at a 50 × 50‐m bin size. Backscatter imagery was processed using QPS Fledermaus FMGT software. As there was very little overlap in the cruise ship tracks, the backscatter was normalized between the two cruises, so that in the regions where there is slight overlap the backscatter intensity is nearly the same. There is a slight difference in normalization noticeable in the center transform valley where the two cruise tracks are juxtaposed, where MGL16‐02 has a higher intensity (Figure 2). In addition, some dropouts for pings are visible due to synchronization between the multibeam and subbottom profiler systems.

Analysis and interpretation of the bathymetry and backscatter were performed using ArcGIS. Within ArcGIS we also calculated the aspect and gradients of the topography to aid analysis of fault scarps, and their orientations. Fault scarps were picked by hand, using a combination of high backscatter intensity to indicate scarps or gradients from steep slopes, bathymetric profiles, and common aspect (plots of the slope direction). The regions of the active ridge were determined by mapping the region of high backscatter intensity, which we infer to be strong backscatter from fresh basalt flows in the topographic lows of the median valleys (Figure 2).

### 2.2 Magnetics

Total field magnetic data were collected on both cruises along the ship tracks. We focused on one nearly W‐E ridge perpendicular profile across the northern ridge segment from MGL16‐02. This is because the entire gridded data set, collected in short nearly N‐S and S‐N ridge parallel tracks, appears to have artifacts owing to a ship track spacing that was broad compared to the scale of the magnetic anomaly. We did not collect a long profile along the southern ridge flanks. To calculate the magnetic anomaly, we removed the International Geomagnetic Reference Field from each data point (Thébault et al., 2015). To account for diurnal variation, we fit a sixth‐order polynomial as a function of time to the data and removed it. We experimented with both lower‐ and higher‐order polynomials and recovered the same result within ±4 nT over the length of the profile.

We inverted the magnetic data for magnetization to aid interpretation using a Fourier Transform method (Parker & Huestis, 1974) because our study location is at relatively low magnetic latitude, with an inclination of −30°, and a declination of −11° (Thébault et al., 2015). We assumed a 500‐m‐thick magnetized layer that followed the bathymetry along the profile, and a magnetization direction that is consistent with a geocentric dipole. We kept the present‐day inclination of −30°, which produced symmetric results near the ridge. We applied a low pass filter to the inversion at 6‐km wavelength, with a 2‐km falloff to stabilize the inversion. We did not include an annihilator to change the mean level of the magnetization.

To improve the age model for the region, we compared our magnetization to the stretched Geomagnetic Polarity Time Scale (GPTS; Cande & Kent, 1995). We applied a 10‐km‐wide Gaussian filter to the GPTS, to mimic the effects of slow accretion at the mid‐ocean ridge and the averaging effects of multiple lava flows of different ages contributing to the magnetization (Schouten & Mccamy, 1972). Using forward modeling, we then varied the apparent spreading rate along the profile in order to match the positive and negative sections of the magnetization to the corresponding GPTS. The stretched GPTS then provided the new age model for the seafloor.

### 2.3 Gravity

The Free Air Gravity anomaly (FAA) was measured on both MGL16‐02 and DY072 using a Bell BMG3 gravimeter and a Lacoste Romberg gravimeter, respectively, at a sample rate of 1 s. Gravity ties were performed before and after both cruises and the data corrected for drift over the duration of the cruises. The data were Eötvös corrected, and then smoothed with a 300‐s moving average filter to reduce the effects of waves and small ship accelerations. We then subtracted the International Gravity Formula from the data. Prior to gridding, time periods where the ship was turning, or accelerating, were removed from the data set. The crossover errors for the surveys and the satellite derived free air gravity anomaly (Sandwell et al., 2014) were < 5 mGal. The final FAA data were gridded along with the satellite derived free air anomaly data to minimize interpolation (Sandwell et al., 2014). We used the Generic Mapping Tools (Wessel & Smith, 1991) splines under tension routine for interpolation, with slightly higher weight being given to the shipboard data (1.5 vs. 1).

We calculate the Mantle Bouguer Anomaly (MBA) from the FAA by subtracting the predicted gravity for the gridded multibeam bathymetry data and a constant thickness crust using the method of Parker (1973). We set the unmapped areas to the median value of the depth across the region. We assumed a density structure of 1,030 kg/m^3^ for water, 2,800 kg/m^3^ for the crust, and 3,300 kg/m^3^ for the mantle with a 6‐km‐thick crust. We neglected the effect of sediments as they are typically <100 m across the region in the global compilation (Divins, 2003; Whittaker et al., 2013), and in estimations from our subbottom profiler data (Agius et al., 2018).

The residual MBA (rMBA), further corrects the MBA for the gravity anomaly associated with the predicted thermal structure of the aging lithosphere (Kuo & Forsyth, 1988). To calculate the thermal model for the area, we used the software package COMSOL to model mantle flow and temperature after Behn et al. (2007). We used the geometry of the ridge‐transform‐ridge section and set up a 500‐km‐wide × 800‐km‐long × 200‐km‐deep model domain. We imposed the half‐spreading rate for the African Plate of 18.2 mm/year, and 15.7 mm/year for the South American Plate at the top of the model space. We allowed open boundaries on all other sides. The thermal boundary conditions included a fixed temperature at the top of 0 °C and 1300 °C at the bottom. The sides of the model had symmetric thermal boundary conditions. We used the same functional form and coefficients for the temperature dependent viscosity structure as Behn et al. (2007). The models were run to steady state. We then translated the thermal structure to density contrast assuming a background density of 3,300 kg/m^3^ and a thermal expansion coefficient of 2.5 × 10^−5^ °C^−1^. The gravity anomaly arising from the thermal density contrast was calculated by summing the upward continued contribution from each 1‐km depth slice in the grid. This anomaly was then subtracted from the MBA to create the rMBA. For 1‐D studies across the ridge, the rMBA was calculated in a similar manner with the same parameters, but the thermal model was calculated from the half‐space cooling model (Parker & Oldenburg, 1973).

We estimated crustal thickness variations from the rMBA. We downward continued the rMBA to the base of the crust approximately 9,000 m below sea level. Then we calculated the variation in topography of the Moho after (Parker, 1973), assuming a density contrast at the Moho of 500 kg/m^3^ (3,300–2,800 kg/m^3^). We then apply a low pass Gaussian shaped filter in the wavenumber domain, with a half‐width of 35 km. We also solved for the lateral density variation of a 6‐km crust to explore the potential effects of serpentinization within the transform system. In this case, we downward continued the rMBA to approximately the midcrustal depth of 6,000 m and assumed a constant thickness layer of 6,000 m to solve for change in density in Parker's equations rather than topography (Parker, 1973).

## 3 Results

### 3.1 Multibeam Bathymetry and Backscatter

The MAR segment to the north and west of the Chain Transform, the Chain Transform, and the MAR to the south and east (Figure 2) can be recognized at a broad scale by the median valley topography and transform fault valley. We describe the detailed morphology visible in the bathymetry and backscatter in each of these regions.

**Figure 3:**
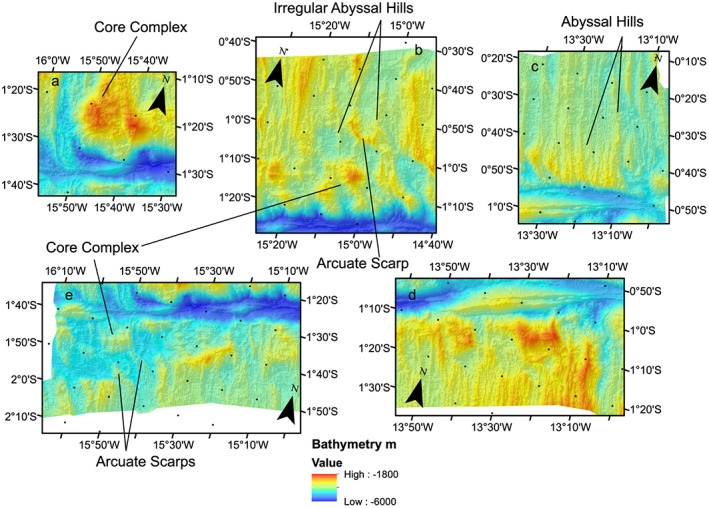
Zoomed‐in view of boxes a–e in Figure 2. Bathymetry is shown by background color and plotted in an oblique Mercator projection. Black dots indicate 10‐arc min graticules.

The active spreading center in the northern MAR segment is visible in the bathymetry as a median valley, deep bathymetry characterized by high backscatter intensity, flanked by shallow bathymetry and moderate backscatter. This character is also typically observed elsewhere on the MAR; the high backscatter valley region is likely associated with fresh basalt (Figures 2a and 2b). The median valley extends directly into the deeper transform area with no offsets, in both the bathymetry and the zone of high backscatter intensity. Normal fault scarps are visible on both sides of the segment. However, on the eastern side within 40 km of the transform into the inside corner high, normal fault scarps are no longer visible and there is a transition to a hummocky seafloor texture (Figures 2, box a, and 3a). This texture is consistent with asymmetric detachment faulting, shorter length, and more irregular abyssal hills and oceanic core complex formation seen in many other segments of the MAR (Escartin et al., 2008). Hereafter, we refer to seafloor with this texture including core complexes, short length irregular abyssal hills, and detachments as *detachment fault* type seafloor and distinguish it from regions with regular normal fault scarps and *abyssal hill* type fabric after (Escartin et al., 2008). On the eastern flank of the ridge this pattern of normal fault/abyssal hill fabric transitioning southward to detachment fault dominated topography extends to 15°18′W longitude. Corrugations are visible on top of some of the shallow blocks suggesting they may be core complexes (Figure 3a).

**Figure 4:**
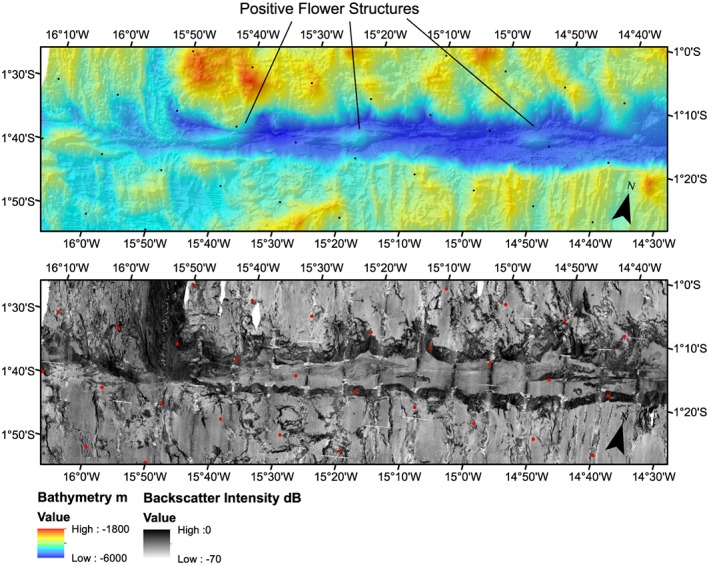
Western Chain Transform, zoomed view. Background color shows bathymetry (top) and grayscale background shows backscatter imagery (bottom). Red and black dots indicate 10‐arc min graticules.

Between 15°18′W and 14°54′W the abyssal hill topography becomes more irregular (Figures 2, box b, and 3b), and a broad basin with flat topography is visible, which is likely sediment filled. Detachment faulting topography is still visible in the south (Figure 4).

Further east of 14.54°W the abyssal hill fabric returns and progressively extends further to the south toward the transform zone going eastward (Figures 3c and 4). The region of detachment faulting dominated topography between the transform zone and the abyssal hill fabric decreases, until 13°30′W (Figures 2, box c, and 3c). The detachment fault topography here is also delimited from the abyssal hill fabric by small arcuate scarps due to small offsets and the formation of a former smaller ridge segment. East of this point the abyssal hill topography abuts and bounds the active transform zone. Further east, detachment faulting topography reappears near the fracture zone.

**Figure 5:**
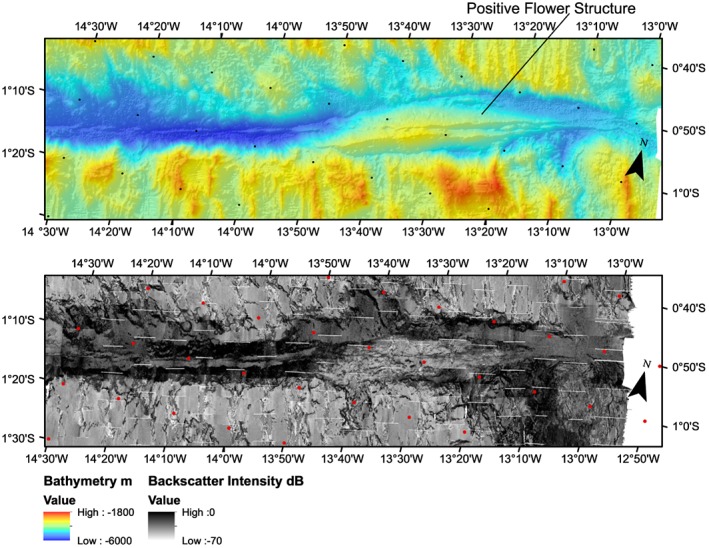
Eastern Chain Transform, zoomed view. Background color shows bathymetry (top) and grayscale background shows backscatter imagery (bottom). Red and black dots indicate 10‐arc min graticules.

The southern MAR segment is identified by the median valley topography and the high‐intensity backscatter. The active ridge segment has a small left‐handed offset going into the transform zone valley indicating a separate ~25‐km‐long ridge segment here. Normal fault topography is visible from south to north until 1°05′S latitude, where the morphology changes to detachment faulting type (Figures 2, box d, 3d, and 5). Going westward, abyssal hill fabric is visible everywhere in the southern parts of our mapped region. Near the transform zone margin the morphology varies between detachment fault and abyssal hill morphologies with little or no evidence for small offsets (Figure 5). The transform margin or valley wall here has high topography and is very clearly defined by a large scarp. Westward from 15°18′W (Figures 2, box e, and 3e), the abyssal hill fabric to detachment fabric transition is offset by left stepping arcuate scarps, with a steep scarp formed at the edge of the abyssal hill fabric. To the west the region of offset detachment faulting fabric broadens, suggesting the small ridge segment decreased in size with time.

We analyzed the abyssal hill fabric to check for rotations, by measuring the orientations of all visible scarps. We found a mean orientation for the region of 347 ± 3°. When analyzed as a function of distance from the ridge, the mean values were all within error of the regional average, suggesting no rotations in spreading direction.

**Figure 6:**
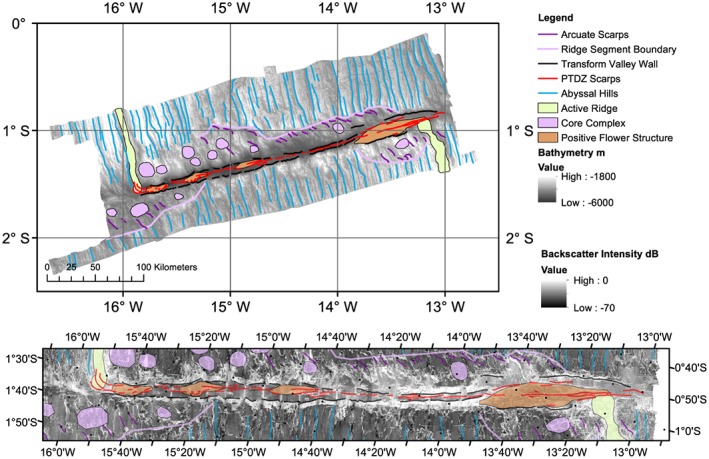
Interpretive map. Interpreted fault morphology over grayscale bathymetry (top) and zoomed interpreted morphology over grayscale backscatter imagery (bottom). Abyssal hill scarps are indicated in blue lines, and presumed principle transform displacement zone (PTDZ) scarps are indicated in red lines. Transform valley wall scarps are indicated in black lines. Ridge segment boundaries are shown as light purple. Small arcuate scarps due to offset are indicated in dark purple lines. The positive flower structures are indicated by orange polygons, the active ridge in light green polygons, and regions with core complexes are indicated in purple polygons. Black dots indicate 10‐arc min graticules.

The Chain transform fault zone ranges from 7 km wide to 20 km wide and extends 300 km from ridge segment to ridge segment (Figures 4, 5, 6). The deepest part of the transform valley is ~5,600 m, while the shallowest portion is ~3,100 m. In contrast to many transforms in the Mid‐Atlantic, the deepest parts of the Chain transform valley are not in the nodal basins (Fox & Gallo, 1984), but rather located in the center of the transform valley. The southern transform wall generally has higher topography, with ~2,000 m of relief and is generally more linear. The scarps have an average trend of 77°. The southern valley wall also maintains its height above the valley to the west relative to the inside corner high, well into the center of the transform. The northern transform wall has a more irregular morphology, in particular, where it is bounded by detachment fault dominated seafloor. In these regions, away from the inside corner high, the height of the bounding scarp above the nearby valley floor is lower, typically 1,000–1,500 m. In the easternmost transform valley at 13°50′W, the northern bounding scarp also offsets to the left, widening the transform zone to it maximum extent of 20 km.

Within the transform valley, major fault scarps are visible (Figures 4, 5, 6) and are likely the principle transform displacement zone (Fox & Gallo, 1984). Most of the fault scarps are linear, trending parallel or subparallel to 77° azimuth, and overlapping in many cases in an en echelon pattern. The longest continuous scarp is mapped in the eastern portion of the transform valley and is 52 km long. Right and left step overs are observed (Figures 4, 5, 6).

**Figure 7:**
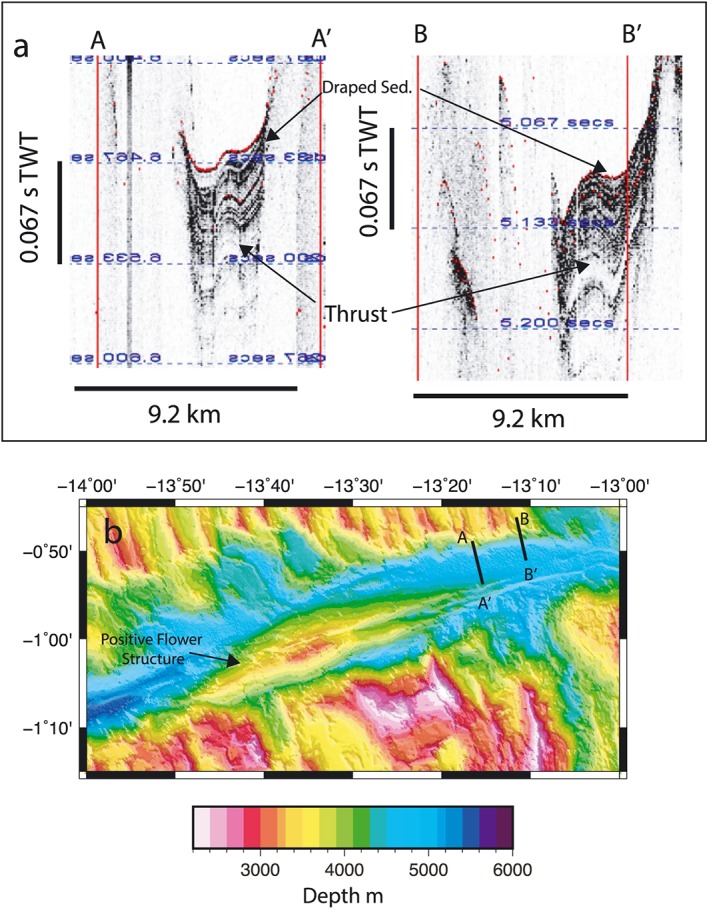
Subbottom profiler transects from MGL16‐02 showing thrusting of sediments at depth and shallower draped sediments. (a) Subbottom profiler transects A‐A' and B‐B′ are indicated on the map (b). Large positive flower structure in the eastern end of the active transform region is visible in to the south and east of the profiles.

There are four positive flower structures visible within the transform as shallow dome‐like structures cut by fault scarps (Figures 4, 5, 6). We interpret these as flower structures based on their high topography, their relationship to the faults surrounding and cutting through them and subbottom profiler data that shows evidence for thrusting in the sediments (Figure 7). The sediments in the subbottom profiler appear to be thrust at depth, but younger sediments appear to be draped, indicating that the large eastern flower structure may not be actively in transpression. In the regions of the positive flower structures the fault scarps tend to offset to the left. The largest positive flower structure is visible in the eastern end of the transform zone (Figure 5), and here the faults can be seen to offset to the left, although the largest fault scarp appears to be cutting though most of the structure, again indicating it may not be in transpression.

### 3.2 Magnetics

**Figure 8:**
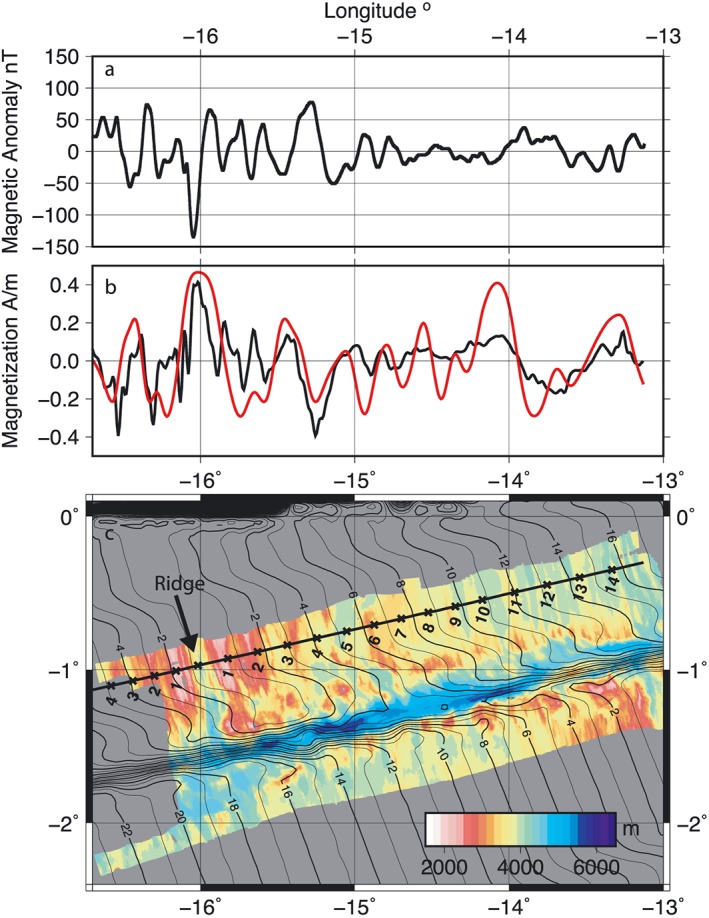
Magnetic anomaly modeling. (a) Total field magnetic anomaly along the profile. (b) Magnetization inversion result (black) and best fit Geomagnetic Polarity Time Scale (red). (c) Isochrons from Müller et al. (1997) shown as black contour lines. Colored background shows bathymetry. Thick black line with crosses shows the location of our profile, with ages from our best fit magnetization model in million years indicated.

The magnetic anomaly along our nearly W‐E ridge perpendicular profile across the northern ridge segment is relatively weak, ranging from −135 to 78 nT, as is expected near the magnetic equator (Figure 8a). The inversion of the magnetization produces low magnetization values, with a maximum of 0.4 A/m (Figure 8b), but with distinct changes in sign that are consistent with paleomagnetic reversals. The inversion produces reasonably symmetric results about the ridge axis. At young seafloor ages, we observe the strongest magnetization, 0.4 A/m, with another peak of similar strength magnetization at 15°30′W. There is one more noticeable peak visible at 13°45′ to 13°12′W that has an amplitude 0.2 A/m.

The stretched filtered GPTS matches the zero crossings of the magnetization record well (Figure 8b), using spreading rates that are within ±6 mm/year of the present‐day spreading rates from the finite pole of rotation for the South Atlantic (Cande et al., 1988). The largest peaks in the magnetization correspond to the present‐day Chron 2 and Chron 5. Comparison of our estimate of the ridge location and age of the seafloor to the global compilation shows that there is a slight error in the location and segmentation of the ridge in the global model for the northern segment; therefore, our local model should provide an improvement (Figure 8c). The location of the southern ridge segment is in good agreement with the global model (Figure 8c), and so the global age model is probably accurate here.

### 3.3 Gravity

**Figure 9:**
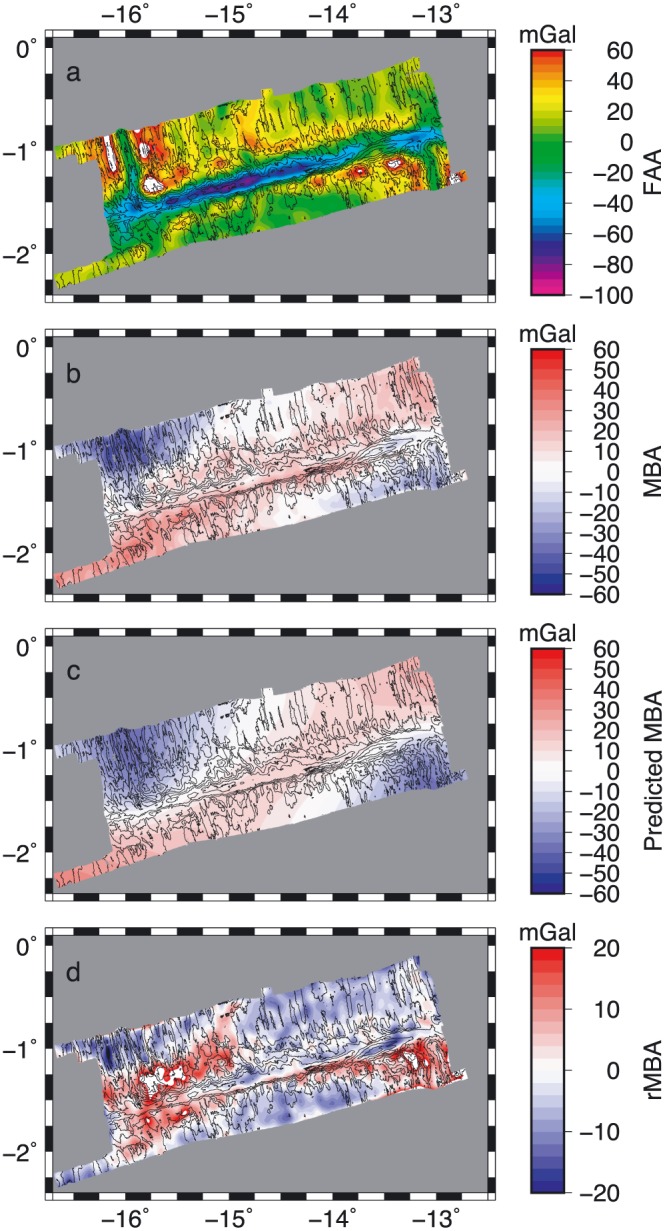
Gravity analysis. (a) Free air anomaly (FAA), (b) Mantle Bouguer Anomaly (MBA), (c) predicted MBA from thermal modeling, and (d) residual MBA (rMBA). Color shows anomaly as indicated. Black lines show 500‐m contours of bathymetry.

The gridded FAA mirrors the bathymetry, with the strongest negatives of −100 mGal in the Chain transform zone, and the most positive values in the shallow regions of the inside corner highs and the ridge segment horsts (Figure 9a). In the MBA map (Figure 9b) there are gravity lows visible along the ridge segments with a minimum of −57 mGal, and the anomaly value increases away from the ridge axes. The largest positive MBA values, 42 mGal, are located on the oldest seafloor. The transform valley and approximately 10 km either side has a slightly positive gravity anomaly of ~5 mGal. A notable exception is the strong negative associated with the large positive flower structure at the eastern end of the Chain transform which has an anomaly of ~ − 13 mGal.

The trend of the MBA with age, increasing away from the ridge, suggests that there is a strong thermal component to the gravity signature. The prediction from the thermal calculation reasonably approximates the pattern observed in the MBA (Figure 9c). Specifically, it matches the trend from the ridges to the oldest seafloor well.

The rMBA (Figure 9d) shows a distinctly negative anomaly of −30–20 mGal focused beneath the center of the northern ridge segment with positive values toward the transform for seafloor west of 15°W (region north of box a, Figure 2). Further east near the region of irregular abyssal hill fabric on the northern segment (Figures 2, box b, and 3b; 3.5‐ to 5.5‐Myr seafloor) there is a negative anomaly that extends from the northern part of the mapped region into the transform zone. Within the transform zone, west of 15°W the rMBA is positive, and to the east it is negative ~ −5 mGal, as low as −10 mGal beneath the eastern positive flower structure. Along the southern ridge segment there is an alternating pattern of positive rMBA on the young age, < 4‐Myr seafloor (Figure 2, box d), negative rMBA from 13°30′ to 15°00′W (4‐ to 17‐Myr seafloor) and then positive rMBA on seafloor >17 Myr (Figures 2, box e, and 3e). In general, the most positive rMBA values are found on the seafloor adjacent to the transform valley, rather than within the transform valley itself.

**Figure 10:**
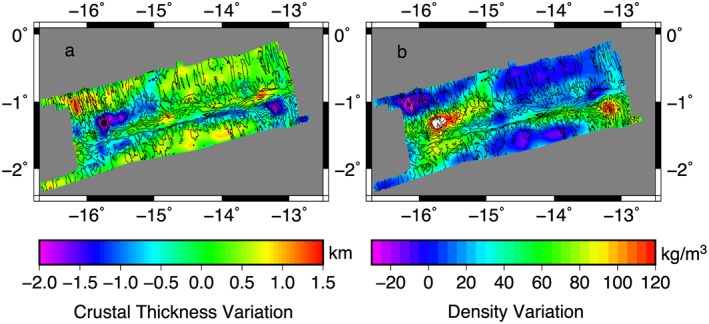
Inferred crustal thickness and density variation from the rMBA. (a) Crustal thickness inferred from the rMBA and (b) Crustal density variation relative to crust from ridge axes inferred from the rMBA. Background color shows crustal thickness or density variation. Black lines show 500‐m contours of bathymetry. rMBA = residual Mantle Bouguer Anomaly.

We calculate the inferred crustal thickness from the rMBA variations (Figure 10a). The thinnest crust can be found where the ridge segments intersect the transform. There are crustal thickness variations in the transform. Within the transform valley, the crust is thinnest in the west by 1 km, but average thickness for most of the transform, particularly beneath the three small flower structures in the west. In the east, near the large positive flower structure, the crust is thicker by up to 1 km. Near the southern valley wall near 14°W the crust is potentially up to 1 km thinner.

**Figure 11:**
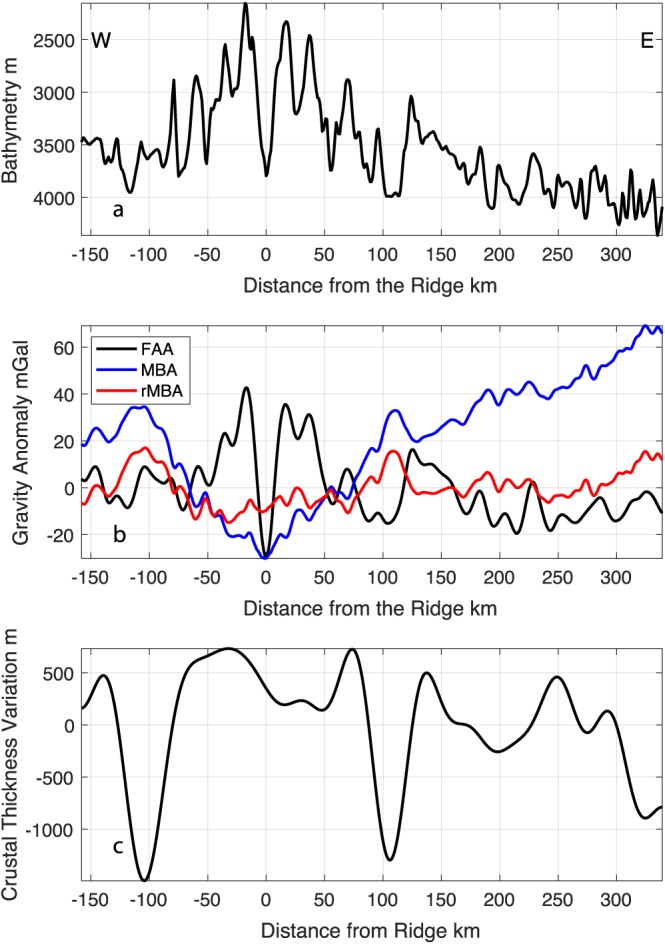
One‐dimensional gravity analysis for conjugate sides of the northern ridge segment. Bathymetry (a) perpendicular to the strike of the ridge is extracted from the 1‐km resolution gridded bathymetry from MGL16‐02 and DY072 cruises; west and east are indicated. The free air anomaly (b) is from satellite gravity (Sandwell et al., 2014), with the MBA and rMBA calculated using the same assumptions used in the 2‐D analysis. Crustal thickness variation from the rMBA (c) shows symmetric thinning on both sides of the ridge at −104‐ and 106‐km distance from the ridge. FAA = free air anomaly; MBA = Mantle Bouguer Anomaly; rMBA = residual MBA.

The inferred crustal thickness is also variable beneath the ridge segments. The thickest crust in the region is found beneath the center of the northern ridge segment. The gravity data suggest that there is slightly thinner crust in the region on the African Plate where the entire ridge segment appears to be in a detachment faulting mode where there is irregular abyssal hill fabric (Figures 2, box b, and 3b). To assess whether this crustal thinning was observed on the conjugate side of the ridge, we calculated the 1‐D rMBA and inferred crustal thickness from our long bathymetric survey lines (Figures 1 and 11). We observe a rMBA high of 16–18 mGal at approximately −100 and 100 km from the ridge. This translates to crustal thinning of 1.5 and 1.3 km respectively, suggesting the thinner crust is a symmetric feature and not due to asymmetric accretion during spreading. On the southern ridge segment, the crust is inferred to be relatively thin, ~2 km (Figure 2, box d), but becomes average thickness from 13°30′W to 15°W. Further west (Figure 2, box e) the crust begins to thin again, ~1 km.

We also translated the rMBA to lateral density variations within the upper 6 km of the crust, assuming that the crust is constant thickness (Figure 10b) to examine what degree of alteration would be required to explain the rMBA. We set the average density of the ridge segment crust to zero, to focus on the variation in the transform valley. Within the transform valley we see evidence for higher densities (up to 90 kg/m^3^) in the west and lower to average densities in the east (−18 kg/m^3^) relative to the average densities of the surrounding crust produced at the ridge segments.

## 4 Discussion

Variations in seafloor fabric and inferred crustal thickness with seafloor age suggest significant changes in melt productivity and tectonism through time in the region. A change from abyssal hill type fabric to detachment fault type morphology is generally associated with a change in the style of crustal formation from magmatically robust to more tectonically dominated mid‐ocean ridge spreading (Blackman et al., 1998; Escartin et al., 2008; Smith et al., 2006). Both the north and south segments exhibit these different styles, although not necessarily synchronously in their histories. This suggests the variations are due to segment‐scale readjustments rather than plate‐scale readjustments. In other words, we would expect variations to occur at similar ages if both the northern and southern segments were being affected by a large‐scale readjustment or a sequential pattern.

The northern ridge segment has had a series of changes in morphology through time, that is, detachment versus abyssal hill fabric, that reflect the evolution of the segment. We discuss the timing using our local magnetic timescale (Figure 8) since there is significant disagreement between our estimate for the location of active ridge and the global seafloor compilation (Müller et al., 1997). From 12.5 to 14 Ma the northern ridge segment was in a magmatically robust period and existed as a single straight segment extending into the transform. From approximately 5.5–12.5 Ma the northern ridge had two segments, the northern segment was more magmatically robust indicated by the abyssal hill fabric, and a smaller segment in the south up to a maximum 20 km long. The abyssal hill fabric is characterized by a small left lateral offset <5 km, with arcuate scarps leading into the transform. The gravity anomaly and inferred crustal thickness suggest that crustal production was relatively uniform across both segments, with <500 m difference in thickness between the segments (Figure 10a). From 3.5 to 5.5 Ma the northern ridge segment had a period of low magmatic productivity, evidenced by weaker abyssal hill fabric and greater detachment fault morphology along the entire length of the mapped segment. In addition, we infer thinner crust based on gravity analysis along the entire mapped segment relative to older and younger seafloor (Figure 10a). This feature is also visible on the conjugate (western) side of the northern ridge segment in a mapping study performed near the Romanche Fracture Zone (Bonatti et al., 1994) and our long survey line. Thinner crust due to low magmatic productivity on the conjugate (western) side ~104 km from the current northern ridge is also supported from our 1‐D rMBA calculations. From 0 to 3.5 Ma the northern ridge shows strong abyssal hill fabric, with a sharp change to detachment faulting topography within ~30 km of the transform. The gravity anomaly suggests that crustal production is higher in the center of the northern ridge segment and that there is thinner crust beneath the detachment fault dominated part of the segment. The fabric leads directly into the transform, and there is no visible offset, that is, arcuate scarps, between the detachment fault dominated and abyssal hill topography, as there is from 5.5–12.5 Ma, in this time window.

The southern ridge segment has three significant phases visible. Here we constrain timing using the global seafloor age compilation (Figure 8c; Müller et al., 1997). From 15 to 20 Ma the southern ridge has two segments, with a small offset of ~6 km estimated from the arcuate scarps observed near the transition from abyssal to detachment seafloor morphology. The smaller segment adjacent to the transform has a maximum length of 30 km. The crust in the small offset segment is inferred to be thinner than seafloor of the same age along the larger segment from gravity. The southern ridge appears to be a single segment with no offsets from 4 to 15 Ma, with some variation in the morphology between abyssal hill fabric and detachment fault dominated fabric immediately near the transform margin. The crustal thickness inferred from gravity during this time period is relatively constant suggesting the segment was relatively magmatically robust. There is a ~6‐km offset visible in the active ridge, suggesting there is a small segment from 0°52′S to 1°05′S latitude. Small arcuate scarps are visible away from the ridge near this offset from 0 to 4 Ma, suggesting the segment has existed throughout this period. The crustal thickness of the segment is thinner than the older seafloor suggesting crustal production may not be as robust near the transform.

Periods of increased magmatism and associated increased crustal production and ridge propagation and segmentation have had an impact on the stress regimes within the transform leading to local transpression. The transpression in turn could have caused crustal thickening by developing the positive flower structures observed within the transform or enhanced serpentinization of the crust and mantle. The southern margin of the transform is remarkably straight on seafloor from 4 to 15 Myr seafloor. The present‐day southern segment appears to have advanced into the transform from 0 to 4 Myr, in the region of the largest positive flower structure. In contrast, the northern margin is irregular, suggesting several ridge segmentations, advances and retreats. In particular, near the large flower structure, the northern transform valley wall offsets to the north going eastward (left lateral offset) between 10 and 11 Ma. The northern ridge advance into the transform zone associated with this offset may have caused the transpression that resulted in the large eastern flower structure around this time. The offsets visible in the boundary faults are well correlated with step overs in the fault scarps within the transform zone itself and with the presence of positive flower structures (Figures 4, 5, 6). Thus, the detailed structure of the fault zone is heavily influenced by the ridge propagation/retreat on either side.

Based on the rMBA, there are likely significant changes in the crustal structure within the transform. It is likely that the crust is thinner immediately adjacent to the northern ridge segment in the west and the southern transform valley wall centered at 14°W based on the positive rMBA, which indicates a mass excess in the region. This is consistent with thinner crust with mantle peridotite at shallower depth. Serpentinization will reduce the density of the mantle by up to 27% for total serpentinization of peridotite (Carlson & Miller, 2003) and so cannot explain a mass excess. However, if present it could reduce the inferred amount of crustal thinning.

Beneath the positive flower structures, the rMBA values are close to the background level of ±5 mGal or are negative beneath the large eastern flower structure. Near the three western flower structures, the rMBA is markedly more positive on the ridge segments on either side and thin crust and/or higher densities are inferred. Similarly, for the largest flower structure in the east the crust on the adjacent southern ridge segment is inferred to be thin. In regions beneath the flower structures the crust could have been thickened relative to the adjacent thin crust of the ridge segments due to transpression, or the density could have been lowered by serpentinization that masks crustal thinning or some combination. Both are likely. Transpression increases crustal thickness. In a similar tectonic setting, serpentinized mylonitic periodities have been recovered from the surface of a positive flower structure in the St. Paul Transform (Maia et al., 2016). We estimate >11–15% serpentinization of the crust and mantle would be needed to produce the ~100‐ to 150‐kg/m^3^ lateral density variation (Figure 10b) we observe across the transform valley (Carlson & Miller, 2003). High‐resolution seismic imaging and rock sampling in the region would be needed to quantify the relative contribution of crustal thickness variation versus serpentinization to the gravity anomaly.

The inferred changes in crustal structure in the transform fault valley contrast with the commonly held view that slow spreading produces thin crust in the mid‐Atlantic transform zones (White et al., 1984). This is normally attributed to reduced magmatic production at the Ridge Transform Intersection, the lack of an intrusive lower crust and serpentinization (White et al., 1984). However, in this region the northern ridge segment appears to have variable magmatic production through time, which may have also influenced the structure within the transform. Transpression may have played a significant role in thickening the crust within the transform. Other studies have found evidence for similarly normal thickness crust in transform zones, with adjacent transform zones <50 km away exhibiting evidence for thinner crust (e.g., Kuo & Forsyth, 1988), highlighting the variability of crustal structure in transforms.

In the broader context of the other large offset transform faults in the equatorial Atlantic, which saw major readjustment and deformation events 10 Ma, the tectonic features in the Chain Transform are muted. Around ~10 Ma the Vema Fracture Zone, saw the construction of its transverse ridge, with a rotation in the strike of the abyssal hill fabric by 10° anticlockwise (Bonatti et al., 2005). The construction of the St. Paul islands and subsequent readjustment appears to have a similar timing (Maia et al., 2016). Further south, the Romanche, also had a failed rotation of the transform by 10° anticlockwise and compressional uplift of a transverse ridge in the east (Bonatti et al., 1994). Along the Chain Fracture Zone there are no transverse ridges and no major reconfigurations of the ridge segments synchronous with the 10‐Ma event. Specifically, we see no evidence for a rotation in the abyssal hill fabric on either of the adjoining segments. The only change visible in the transform valley configuration, which could be consistent with an anticlockwise rotation of ~5°, is the abrupt left lateral step in the northern transform valley wall between 10 and 11 Ma, which may have led to the formation of the large eastern flower structure. The positive flower structures appear to be a function of segment‐scale reorganization rather than a regional scale readjustment. The only major change in ridge magmatic productivity appears to be a weakening of magmatism at 3.5–5.5 Myr, which does not appear to correlate with any observed major deformation in the Romanche. Therefore, the Chain Transform may be toward the southern limit of the 10 Ma event and experienced little associated deformation, just subsequent readjustment of the adjacent spreading centers.

## 5 Conclusions

We present results from a marine geophysical survey of the Chain Transform and the adjacent ridge segments. We find that there are variations with time in the magmatic productivity of the individual ridge segments, inferred from seafloor fabric and gravity analysis. The ridge segments change modes between abyssal hill production and detachment fault production within 40 km of the transform zone. In addition, we find strong evidence for crustal thickening or a substantial amount of serpentinization of the crust inferred from the gravity anomalies and shallow bathymetry within the active transform zone, mostly in the form of a large positive flower structures. The variations in crustal thickness or serpentinization and ridge segmentation, magmatic productivity, and transpression within the transform do not appear to be associated with a significant deformation event at 10 Ma. observed at large offset transforms further north along the Mid‐Atlantic Ridge. The only deformation event that may have been related to this event is the formation of the large eastern positive flower structure, which was produced by a local ridge segment adjustment. This suggests the Chain Transform may be the southern extent of these deformation events.
